# Dose-Limiting Toxicities of Paclitaxel in Breast Cancer Patients: Studying Interactions Between Pharmacokinetics, Physical Activity, and Body Composition—A Protocol for an Observational Cohort Study

**DOI:** 10.3390/cancers17010050

**Published:** 2024-12-27

**Authors:** Len De Nys, Anita Barzegar-Fallah, Katrien Lanckmans, Stephane Steurbaut, David Beckwée, Amy de Haar-Holleman, Steven Provyn, Elke Gasthuys, Sofie Vande Casteele, Pieter-Jan De Sutter, An Vermeulen, Jan Van Bocxlaer, Stephanie C. M. Wuyts, Nele Adriaenssens

**Affiliations:** 1 Rehabilitation Research, Vrije Universiteit Brussel (VUB), Laarbeeklaan 121, 1090 Jette, Belgium; len.de.nys@vub.be (L.D.N.);; 2 Medical Oncology Department, Universitair Ziekenhuis Brussel (UZ Brussel), 1090 Jette, Belgium; 3 Clinical Biology Department, Universitair Ziekenhuis Brussel (UZ Brussel), 1090 Jette, Belgium; 4 Pharmacy Department, Universitair Ziekenhuis Brussel (UZ Brussel), 1090 Jette, Belgium; stephane.steurbaut@uzbrussel.be (S.S.);; 5 Vitality Research Group, Vrije Universiteit Brussel (VUB), 1090 Jette, Belgium; 6 Translational Oncology Research Center, Vrije Universiteit Brusse (VUB), 1050 Brussels, Belgium; 7 Human Physiology and Sports Physiotherapy (MFYS), Vrije Universiteit Brussel (VUB), 1090 Jette, Belgium; 8 Department of Bioanalysis, Faculty of Pharmaceutical Sciences, Ghent University, 9000 Gent, Belgium; elke.gasthuys@ugent.be (E.G.);; 9 Research Centre for Digital Medicine, Vrije Universiteit Brussel (VUB), 1090 Brussels, Belgium

**Keywords:** breast cancer, chemotherapy, toxicity

## Abstract

**Background/Objectives**: Paclitaxel (PTX), a commonly used chemotherapy for breast cancer (BC), is associated with dose-limiting toxicities (DLTs) such as peripheral neuropathy and neutropenia. These toxicities frequently lead to dose reductions, treatment delays, or therapy discontinuation, negatively affecting patients’ quality of life and clinical outcomes. Current dosing strategies based on body surface area (BSA) fail to account for individual variations in body composition (skeletal muscle mass (SMM) and adipose tissue (AT) mass) and physical activity (PA), which can influence drug metabolism and toxicity. This study aims to explore the relationships between PTX pharmacokinetics, body composition, and PA to predict DLTs. **Methods**: This single-group observational cohort study will recruit 40 female BC patients undergoing PTX treatment. Data collection will include plasma PTX concentrations, body composition assessments (using dual X-ray absorptiometry and bioelectrical impedance analysis), PA measurements (via accelerometers), and questionnaires to assess BC-related health-related quality of life, chemotherapy-induced peripheral neuropathy, and neutropenia during the PTX schedule using validated questionnaires. Dose-limiting toxicities will be graded according to the Common Terminology Criteria for Adverse Events v5.0 (grade 3 or higher). This protocol is designed to develop a population-based PK-PD model that predicts the occurrence of chemotherapy-induced peripheral neuropathy and neutropenia in women with stage II or III BC undergoing PTX therapy, focusing on explanatory outcomes related to SMM, AT mass, and PA.

## Simple Summary

Breast cancer treatment with the chemotherapy drug paclitaxel (PTX) is highly effective. Still, it can cause serious side effects, such as nerve damage (peripheral neuropathy) and a reduced ability to fight infections (neutropenia). These side effects lead to dose reductions or delays in treatment, which can impact patients’ quality of life and the effectiveness of their care. Current dosing methods are based on body surface area but do not consider individual differences in muscle and fat levels or physical activity, which can influence how the drug is processed and tolerated. This study will explore how these factors interact with paclitaxel and contribute to side effects. We aim to develop a model that predicts these effects in women with breast cancer. This may help refine chemotherapy dosing and reduce side effects in the future.

## 1. Introduction

Background: Breast cancer (BC) treatment with paclitaxel (PTX) is often complicated by dose-limiting toxicities (DLTs), which can impact treatment efficacy due to relative dose intensity adaptations (RDI) [1,2]. PTX is a widely used and efficacious taxane cytotoxic chemotherapeutic agent [3,4,5]. It stabilizes microtubules, which inhibits cancer cell division and induces cell death [6]. However, it also affects healthy dividing cells, potentially leading to adverse effects such as peripheral neuropathy, neutropenia, and hematological, gastrointestinal, and cardiac adverse effects [1,7,8]. Among these toxicities, chemotherapy-induced peripheral neuropathy (CIPN) and neutropenia are primary concerns [6,8] as they affect a majority of patients and often persist long-term. These toxicities can significantly impair health-related quality of life (HRQoL) [8,9,10,11]. In addition, DLTs can result in treatment delays, dose reductions, or even premature termination of therapy that can compromise clinical outcomes [12,13,14,15,16,17].

Mechanisms and Limitations of Current Dosing Approaches: The dosing of PTX is primarily based on body surface area (BSA), an approach initially intended to standardize dosing across patients with varying body sizes [18]. However, BSA has limited utility in accounting for inter-individual pharmacokinetic (PK) and pharmacodynamic (PD) variability, especially in patients with diverse body compositions [19]. BSA-based dosing does not effectively reflect body composition metrics, such as skeletal muscle mass (SMM) and adipose tissue (AT), which may influence PTX distribution, metabolism, and clearance, thereby contributing to variability in treatment outcomes and the risk of DLTs [20,21,22]. Recent studies suggest that body composition, rather than BSA, may provide more accurate PK and PD predictions, especially in chemotherapy dosing [23,24]. This highlights a need to re-evaluate dosing practices to better account for individual patient characteristics.

Existing Knowledge on Body Composition and Physical Activity in Chemotherapy Tolerance: Evidence indicates that in patients with breast cancer, low SMM and high AT are associated with increased susceptibility to PTX-induced DLTs [13,21,23,25,26,27]. To illustrate, BC patients with low lean body mass, such as those with sarcopenia (a condition characterized by the loss of skeletal muscle mass and strength) or sarcopenic obesity (the coexistence of sarcopenia with excess body fat), are at a higher risk of increased drug toxicity due to altered drug distribution and clearance [13,28,29,30], potentially leading to side effects like CIPN [20]. Given PTX’s hydrophobic nature and its tendency to partition in AT [19], understanding the relationship between body composition and drug toxicity is crucial for optimizing dosing regimens [20,24].

Additionally, physical activity (PA), an often-underutilized therapeutic factor in oncology, has been shown to mitigate chemotherapy toxicities, including fatigue, pain, and CIPN, and improve the quality of life in cancer patients [31,32,33,34]. Studies have also suggested that PA may modulate chemotherapy pharmacokinetics, potentially influencing PTX tolerance, by enhancing physiological processes such as increased hepatic and renal blood flow, which are critical for drug metabolism and elimination, thereby impacting treatment efficacy [31,34,35,36,37,38,39]. Despite these findings, there is limited research specifically examining the combined effects of body composition and PA on PTX toxicities in BC patients.

Need for the trial: Given the limitations of current dosing practices and the observed variability in PTX-related toxicities, there is a pressing need for research that integrates body composition and PA parameters into PTX dosing strategies to better predict and mitigate toxic responses. This study addresses this need by investigating how body composition, i.e., SMM and AT mass, and PA-levels influence PTX-induced DLTs in BC patients. The study combines established methodologies—pharmacokinetics, body composition assessments via DXA/BIA, and accelerometry for PA—which have individually demonstrated value in oncology research. However, their integrated application to predict PTX-induced toxicities remains underexplored. This study builds on evidence linking body composition and activity levels to chemotherapy outcomes [13,15,17,30,34,35], providing a foundation for this and future investigations. This trial is registered as NCT06387901 (clinicaltrials.gov).

Study objectives: It is hypothesized that lower SMM and reduced PA levels in BC patients are associated with a higher incidence and severity of PTX-induced DLTs. Furthermore, a PK-PD model will be developed to predict individual patient responses and DLTs.

Primary objectives

- To develop a population-based PK-PD model that predicts the occurrence of CIPN in women with stage II or III BC undergoing PTX therapy, focusing on explanatory outcomes related to body composition (SMM and AT mass) and PA.

Secondary objectives

- To evaluate PTX exposure by measuring plasma concentrations, specifically assessing peak plasma concentration (Cmax) and the duration above a threshold concentration of 0.05 µmol/L (time above 0.05 µmol/L).
- To assess the frequency, types, and severity of DLTs, generally defined as clinically relevant toxicities grade 3 or higher according to the Common Terminology Criteria for Adverse Events (CTCAE) classification v5.0.
- To track changes in body composition, with particular emphasis on SMM and AT mass, throughout PTX therapy.
- To explore the relationships between body composition, PA, PTX plasma levels, and the incidence and severity of DLTs during PTX treatment.
- To further refine the PK-PD model by examining the impact of the studied parameters and relevant covariates on PTX exposure, aiming to determine the optimal PTX dose for individual BC patients based on these insights.
- To assess BC-related HRQoL, CIPN symptoms and neutropenia during the PTX schedule using validated questionnaires.

## 2. Methodology

### 2.1. Methods: Participants, Interventions

#### 2.1.1. Study Setting

The study will be conducted at the Medical Oncology Department of UZ Brussel, Belgium, where data collection and clinical assessments will occur. Patient screening is coordinated through the ‘Multidisciplinary Oncological Consult Breast’ meetings of the Medical Oncology Department, held weekly at UZ Brussel, ensuring the streamlined identification and recruitment of eligible participants.

#### 2.1.2. Eligibility Criteria

Inclusion Criteria:

Participants eligible for this study must meet the following criteria:

- Female patients aged 18 years or older (pre- or postmenopausal).
- Diagnosis of stage II or III BC.
- Scheduled to receive 12 weekly PTX cycles in a (neo-)adjuvant setting (once-a-week schedule). Concomitant targeted therapy is allowed if no overlapping toxicity profile.
- Previous taxane exposure is permitted, provided the last treatment was completed more than one year before enrollment.

Exclusion Criteria:

- Cognitive impairment that may interfere with understanding or completing study assessments.
- Current enrollment in clinical trials involving experimental drugs.
- Documented intolerance or allergy to PTX; participants with non-documented intolerance or allergy will be withdrawn if symptoms arise.
- Concurrent use of medications known to interact with PTX or potentially interfere with study assessments.
- Ongoing chemotherapy-induced peripheral neuropathy (CIPN) from previous treatments; any CIPN must be resolved prior to study entry.
- Presence of diabetes with peripheral neuropathy due to potential confounding effects on study assessments.

#### 2.1.3. Intervention

In the study, PTX will be administrated according to standard clinical practice at a dose of 80 mg/m^2^, via intravenous infusion, on a weekly schedule. Blood samples, body composition assessments, and PA measurements will be collected at designated intervals to analyze PK and PD parameters.

#### 2.1.4. Modifications

In the event of adverse events or intolerance to PTX, participants’ treatment may be adjusted or interrupted at the discretion of the treating medical oncologist, with modifications documented in the study records. Compliance with PTX depends on the DLT. As a proxy for treatment compliance, relative dose intensity (RDI) will be calculated, following the definitions in Appendix A Table A1. RDI is used as a surrogate measure of effectiveness by calculating the proportion of administrated dose per m^2^ of body surface area per week in relation to the optimal dose per m^2^ of body surface area per week [40,41]. If a PTX cycle is postponed, assessments are postponed as well; if a PTX cycle is skipped, assessments are skipped as well. Participants can withdraw from the study at any time without impacting their standard medical care.

#### 2.1.5. Adherence and Monitoring

Participant adherence to protocol assessments is supported by direct engagement with oncology staff, reminders for assessment visits, and the involvement of a research coordinator to ensure compliance.

#### 2.1.6. Concomitant Care

Participants may continue standard treatments and medications as prescribed by their oncologist. Medications known to interfere with PTX pharmacokinetics are prohibited to maintain data integrity for PK-PD analyses.

### 2.2. Methods: Outcome Data Collection, Management, and Analysis

Data will be collected at specified time points during and after PTX therapy. The collection methods are structured to capture comprehensive data across primary and secondary outcomes, ensuring high data quality and reliability (see Table 1: Participant timeline). Training sessions for study staff will emphasize data collection accuracy, equipment use, and procedures, and protocols for each assessment will ensure standardized implementation. All forms and materials are securely stored in the REDCap^®^ database (Vanderbilt, Nashville, TN, USA), where audit trails monitor access and edits.

#### 2.2.1. Primary Outcome

The primary objective is to develop a population PK model that describes the PK of PTX in plasma. The development of such a compartmental model will be conducted, starting from models described in the literature [13]. The inter-individual variability in PK parameters will be estimated directly from the data and the potential effects of covariates (e.g., age, weight, creatinine clearance, laboratory values, race, …), primarily on PTX clearance, will be determined. Special focus will be placed on body composition parameters such as SMM and AT mass, as well as PA to evaluate their impact on the PK variability of PTX. Next, a population PK-PD model will be developed to link PTX exposure with safety outcomes in individual patients, particularly focusing on PTX-induced peripheral neuropathy and potentially also neutropenia.

#### 2.2.2. Secondary Outcomes

##### PTX Exposure

PTX exposure will be measured through two specific plasma concentration metrics: maximum plasma concentration (Cmax) and the time during which plasma levels remain above a threshold concentration of 0.05 µmol/L (Tc > 0.05). This threshold was chosen based on previous studies showing that PTX plasma concentrations above 0.05 μmol/L (42.7 ng/mL) are predictive for DLTs in BC patients [42]. A study nurse will collect blood samples during cycles 1 (T1), 6 (T2), 9 (T3), and 12 (T4) at two precise time points:

- First Sample: Collected within 10 min of post-infusion start at the hospital to determine Cmax.
- Second Sample: Collected 16 to 26 h post-infusion at the patient’s home to calculate Tc > 0.05.

Two methods will be used for blood sampling: dried blood spots (DBS) for all participants and additional standard blood collection tubes for the first 10 participants. DBS samples will be collected from the non-dominant hand and dried on PerkinElmer 226 Bioanalysis RUO Cards (Revvity Health Sciences Inc GR2261004, Revvity Health Sciences B.V., Rigaweg 22, 9723 TH Groningen, The Netherlands). These DBS samples will then be stored in a −80 °C freezer. Blood concentrations obtained from DBS are not equivalent to plasma concentrations, as the partitioning of PTX between blood and plasma may vary. For this reason, the relationship between blood and plasma concentrations will be investigated in the subset of the first ten patients, allowing the estimation of plasma concentrations from DBS-derived blood concentrations in the remaining participants. This approach will enable accurate PK-PD modeling based on (extrapolated) plasma concentrations.

Plasma samples collected via tubes for the first 10 participants will be centrifuged within 3 h of collection at 2000× *g* for 10 min at room temperature. The resulting plasma will be divided into two portions, each transferred to separate cryotubes—one for analysis and one as a backup. Both cryotubes will be stored immediately at −80 °C. All samples will be transported in a temperature-controlled mobile unit (Sterling ultracold ULT25NEU, Athens, OH, USA) to the analytical lab (Laboratory of Medical Biochemistry and Clinical Analysis, Ghent University, Gent, Belgium).

##### Dose-Limiting Toxicities

DLTs will be recorded after each assessment point (T1, T2, T3, T4) and graded according to the National Cancer Institute’s Common Terminology Criteria for Adverse Events (CTCAE v5.0, NIH, National Cancer Institute, Cancer Therapy Evaluation Program). Common DLTs such as CIPN and neutropenia will be closely monitored, with neutropenia assessed via regular blood tests and CIPN tracked through patient-reported outcomes.

Additional adverse events, including dose modifications, treatment delays, interruptions, and unplanned hospital visits, will also be recorded to provide a comprehensive profile of treatment tolerability.

##### Body Composition Changes

The integration of body composition assessment is essential for large-scale clinical workflows in oncology [43]. Body composition changes during PTX therapy will be tracked using Dual X-ray Absorptiometry (DXA, Hologic QDR 4500, Hologic, Inc., headquartered at 35 Crosby Drive, Bedford, MA, USA) and Bioelectrical Impedance Analysis (BIA, InBody S10, InBody Co., Ltd., headquartered in Seoul, Republic of Korea) at baseline (T0) and before PTX infusions during cycles T2 and T4. Key metrics will include absolute and relative measures of SMM and AT, providing insights into how these factors fluctuate throughout treatment.

Both DXA and BIA are fast, non-invasive, cost-effective, and feasible methods for quantifying the components of body composition. They have demonstrated validity and reliability [44], providing comprehensive total body data and regional insights, including on trunk, arms, legs, pelvis, and android/gynoid regions [45]. While DXA is regarded as the gold standard for measuring MM and AT mass, BIA effectively quantifies total body water, extracellular fluid, and intracellular fluid.

##### Physical Activity Levels

To objectively measure PA levels, participants will wear an Actigraph GT3X+ accelerometer on the right hip for seven days following PTX infusions at cycles T1–T4. The device, water-resistant and comfortable for continuous wear, will track energy expenditure, activity intensity (low, moderate, vigorous), and sedentary behavior.

PA data will be processed in ActiLife software (version 6.11.9, ProCare), aggregating activity counts into 60 s epochs. Outcome measures will categorize PA into low (≤1951 counts/min), moderate (1952–5724 counts/min), and vigorous (≥5725 counts/min) intensity, based on the cut-off points of Freedson [46], with the total daily PA calculated as the sum of all intensities.

PA levels will also be evaluated using the European Health Interview Survey-Physical Activity Questionnaire (EHIS-PAQ) [47]. This self-reported questionnaire provides a comprehensive view of participants’ PA across various domains, including leisure, transport, and occupational activities. The EHIS-PAQ data will complement the objective Actigraph measurements by capturing types and contexts of PA that may not be detected through accelerometry alone, offering a holistic assessment of participants’ PA behaviors during the study.

##### Correlation Analyses

Correlation analyses will explore associations between body composition metrics (SMM and AT mass), PA levels, plasma PTX concentrations, and DLT incidence/severity. These analyses aim to identify key predictors of adverse outcomes and validate the PK-PD model by confirming the relevance of specific parameters in DLT prediction.

##### Health-Related Quality of Life

HRQoL will be evaluated using the European Organization for Research and Treatment of Cancer Quality of Life Questionnaire (EORTC QLQ-C30) [48] at baseline (T0) and during follow-up after cycles T2, T3, and T4. Additional modules include the following:

- EORTC QLQ-BR45 [49]: A BC-specific module assessing BC-related symptoms and issues relevant to patient quality of life during treatment.
- EORTC QLQ-CIPN20 [50]: A CIPN-specific module assessing the impact of neuropathy on patient quality of life.

Each questionnaire provides scores across multiple symptom scales (e.g., fatigue, pain, sleep disturbances) and functional domains, with high responsiveness and validity for detecting quality-of-life changes during PTX therapy.

##### Exploratory Outcomes

The following data will be obtained from participants’ medical files for explorative analyses: (1) age, height, and weight; (2) tumor type (hormone sensitivity and HER2); (3) relevant comorbidities, including diabetes mellitus, multiple sclerosis, polyneuropathy, Chronic Obstructive Pulmonary Disease (COPD), kidney and/or liver disease, and cardiac disorders; (4) (neo-)adjuvant/concomitant treatment; (5) race; (6) ethylism; (7) albumin (g/dL); (8) liver function tests (ASAT (U/L), ALAT (U/L), GGT (U/L), total bilirubin (mg/dL), renal function (creatinine (mg/dL), and eGFR (mL/min/1.73 m2); (9) comedications or interacting drugs with similar toxicity profiles; (10) cryotherapy (gloves, socks, and/or cap) during PTX infusion.

#### 2.2.3. Participant Timeline

The participant timeline is as follows (also detailed in Table 1): (1) Before the initiation of PTX treatment, each participant undergoes an eligibility screening. Those who meet the criteria are provided with detailed study information and, upon agreement, complete the informed consent process. (2) Baseline data collection includes gathering demographic information and clinical history. (3) Throughout the PTX treatment at cycles 1, 6, 9, and 12, participants undergo multiple assessments. Blood samples are collected for pharmacokinetic analysis, body composition is evaluated, and DLTs are monitored. Physical activity is tracked using accelerometers, capturing data on daily movement patterns. (4) Approximately one week after the final PTX cycle (T4), participants complete a follow-up assessment. This includes a final DLT evaluation, body composition measurement, and PA monitoring to assess any changes following the completion of treatment.

#### 2.2.4. Sample Size

The study will enroll 40 female BC patients to ensure sufficient data despite potential attrition (anticipated at 20%). To mitigate the risks associated with a small sample size, the study requires that at least two patient groups (RDI> or ≤85%) are distinguishable by the 12-week mark, as dose reductions exceeding 15% have been shown to significantly predict poorer overall survival [51]. This approach is supported by findings from comparable PK studies evaluating PTX, showing the importance of sufficient observations to draw meaningful conclusions [13]. Given the study’s exploratory nature, future studies will refine sample size calculations based on the findings of the present study.

#### 2.2.5. Recruitment

Participants are identified during weekly ‘Multidisciplinary Oncological Consult Breast’ meetings at UZ Brussel and approached by the oncologist and/or the research team directly to discuss study involvement. Recruitment will continue until the target sample size is achieved, with dedicated research staff supporting participant engagement and follow-up.

### 2.3. Methods—Data Management, Analysis, and Monitoring

#### 2.3.1. Data Management

Double data entry with range checks is implemented in REDCap^®^ [52,53]. Data consistency checks and regular audits will verify data accuracy. REDCap^®^ will securely house electronic data with access restricted to authorized personnel. Local and institutional servers (Microsoft SharePoint^®^) will host synchronized copies, with automated daily backups. Data will be stored on encrypted devices, with research data preserved for 10 years and clinical data for 25 years, following Belgian data retention laws and institutional policy.

#### 2.3.2. Statistical Analysis Plan

##### Primary Outcome

Logistic regression models will be used to describe the occurrence of CIPN, while a semi-mechanistic model will be employed to describe neutropenia [54]. Finally, nonlinear mixed-effects modeling will be performed using Monolix^®^ software (Version number: 2024R1, Lixoft SAS, Antony, France) and the SAEM algorithm to develop the PK-PD model for DLT prediction based on body composition and PA parameters.

##### Secondary Outcomes

Descriptive and inferential statistics using IBM SPSS (Version number: 30, IBM, Corp., Arming, NY, USA) will assess DLT incidence, PTX exposure metrics, and body composition changes. For correlation analysis, mediation analysis will determine the relationships between PK, body composition, PA, and DLTs.

##### Additional Analyses

Subgroup analyses will investigate the effects of covariates such as age, baseline PA levels, and body composition on DLT outcomes. Adjusted analyses will control for potential confounders, including comorbidities and concurrent treatments.

##### Population Definition and Missing Data

The study population includes all enrolled participants who complete at least one follow-up assessment. Participants who discontinue PTX treatment early or are lost to follow-up will be retained in analyses using imputed data, where feasible, to maximize statistical power and limit bias. For missing data, multiple imputation methods will be used. Sensitivity analyses will be conducted to examine the robustness of outcomes, exploring both complete case analyses and imputed datasets.

#### 2.3.3. Data Monitoring

A formal Data Monitoring Committee is not deemed necessary for this observational study, as it lacks investigational medicinal products. Instead, the Trial Steering Committee (TSC) and an Expert Advisory Board will serve a similar oversight function. The TSC comprises members of the Trial Management Group, work package leaders, two pharmacists from UZ Brussel, and two patient representatives from the Patient Advisory Board. The Expert Advisory Board comprises two oncologists, an expert in clinical research in oncology, an expert in PK-PD modeling and two innovation and valorization managers. The TSC’s and Experts Advisory Board’s primary role includes monitoring trial progress, advising on scientific and ethical issues, ensuring adherence to protocol, and making shared decisions. It is independent of the study funder (Kom op tegen Kanker), with members holding no competing interests.

An interim analysis will be conducted following data collection from the first 10 participants. This analysis will assess early trends in safety, pharmacokinetics, and outcomes related to body composition and PA. The TSC will review interim results to determine if any adjustments are required. The TSC also holds the final authority to terminate the study if necessary.

#### 2.3.4. Harms

As the trial is observational, adverse events associated with PTX are well characterized, with no additional safety reporting beyond standard care requirements. However, adverse events will be captured as outcomes. The study design includes all adverse events, especially DLTs like CIPN and neutropenia, which will be monitored and recorded using the CTCAE v5.0, and all treatment modifications (e.g., dose delays, interruptions) will be recorded to capture any impact on study conduct or outcomes.

#### 2.3.5. Auditing

The trial will undergo regular internal audits by TSC members, specifically work package leaders and quality control personnel from UZ Brussel. These audits will verify adherence to protocol, data accuracy, and ethical compliance. External audits by regulatory authorities or the local institutional review board may also be conducted if deemed necessary.

## 3. Ethics and Dissemination

### 3.1. Research Ethics Approval

The study received ethical approval from the Medical Ethics Committee of UZ Brussel (reference BUN: 1432024000096). The protocol adheres to the Declaration of Helsinki principles, ensuring ethical conduct and participant rights protection throughout the study.

### 3.2. Protocol Amendments

Any significant protocol modifications, such as changes in eligibility criteria, outcomes, or analyses, will be communicated to all relevant stakeholders, including the institutional review board, investigators, participants, trial registry, and any other required regulatory bodies. Modifications will also be reflected in all relevant study documentation and reports.

### 3.3. Informed Consent

Informed consent will be obtained by medical oncologists from UZ Brussel’s Medical Oncology Department. During consultations, oncologists will inform eligible patients about PTX therapy and this observational study. Interested participants will sign an informed consent form before any study procedures. Consent forms in Dutch, French, and English are available and have been approved by the Medical Ethics Committee (the corresponding author will provide copies of the informed consent forms upon reasonable request, ensuring compliance with ethical and privacy guidelines).

### 3.4. Confidentiality and Data Protection

Participant confidentiality will be protected through data pseudonymization. Participants will be assigned a unique study code, and the Subject Identification Log, linking codes to identifiable information, will be accessible only to authorized study staff. Patient data will be stored securely in REDCap and VUB SharePoint, with encryption and password-protected access. All sensitive information (e.g., consent forms, communication) is encrypted and stored on VUB SharePoint, a secure, encrypted infrastructure. In line with the General Data Protection Regulation (GDPR), participant data will be retained securely for at least 10 years post-study, and clinical trial data will remain stored for 25 years per Belgian law.

### 3.5. Dissemination

Study findings will be shared transparently with TSC, the Patient Advisory Board, and the Expert Advisory Board. Participants will receive lay summaries of the results, which will appear on institutional websites and social media. Results will be submitted to peer-reviewed journals, preferably with open access, and presented at conferences.

### 3.6. Data Sharing and Accessibility

In adherence to local data protection laws and FAIR principles, de-identified study data will be available upon request to support further research, ensuring compliance with good clinical practices (GCP) and GDPR.

## Figures and Tables

**Table 1:** Participant timeline.

Description of PTX Treatment		Before PTX	PTX for 12 Cycles (12 Weeks)											After PTX
Relationship to Chemo Cycles		∼Weeks Prior (T0)	Cycle 1 (T1)		T1 + 7 Days	Cycle 6 (T2)		T2 + 7 Days	Cycle 9 (T3)		T3 + 7 Days	Cycle 12 (T4)		T4 + 7 Days
			D1	D2		D1	D2		D1	D2		D1	D2	
Informed consent form (ICF)		X												
Inventory of home medication		X				X			X			X		
Baseline data collection			X											
Body weight on scale			X			X			X			X		
DXA scan			X			X						X		
BIA			X			X						X		
Activity tracker			X _a_			X _a_			X _a_			X _a_		
Blood sample	Tube (in hospital)		X _b_			X _b_			X _b_			X _b_		
	DBS (in hospital)		X			X			X			X		
	Tube (at home)			X _b_			X _b_			X _b_			X _b_	
	DBS (at home)			X			X			X			X	
Survey EORTC QLQ-C30			X		X			X			X			X
Survey EORTC QLQ-BR45			X		X			X			X			X
Survey EORTC QLQ-CIPN20			X		X			X			X			X
Survey EHIS PA Q			X		X			X			X			X
Inventory of DLTs by oncologists					X			X			X			X

_a_ The activity tracker will be worn continuously for 7 days, starting from the day of the PTX infusion (day 1). _b_ Only the first ten enrolled patients will need to provide two blood samples: one tube sample and one DBS sample. BIA: Bioelectrical impedance analysis, DBS: Dried blood spots, DLTs: Dose-limiting toxicities, D represents specific days (e.g., D1, D2) within each treatment cycle.

## Data Availability

No new data were created or analyzed in this study. Data sharing is not applicable to this article.

## Appendix A

**Table A1:** Relative dose intensity calculation.

Item	Definition
RDI	(DDI/SDI) × 100%
DDI (delivered dose intensity)	(Delivered total PTX dose, in mg/m^2^)/(actual time to complete chemotherapy with imputation for missed cycles, in days)
SDI (standard dose intensity)	(Standard total PTX dose, in mg/m^2^)/(standard time to complete chemotherapy, in days)
DTD (delivered total dose)	Total amount of PTX actually administered over chemotherapy course
Actual time to complete chemotherapy with imputation for missed cycles	Observed number of days between initiation and final receipt of chemotherapy plus expected number of days for missing cycles
STD (standard total dose)	Total standard amount of drug for administration over chemotherapy course
Standard time to complete chemotherapy	Standard number of days between initiation and final receipt of chemotherapy

RDI: Relative Dose Intensity, DDI: Delivered Dose Intensity, SDI: Standard Dose Intensity, DTD: Delivered Total Dose, STD: Standard Total Dose.
